# Myeloproliferative neoplasms: From JAK2 mutations discovery to JAK2 inhibitor therapies

**DOI:** 10.18632/oncotarget.281

**Published:** 2011-06-05

**Authors:** Francesco Passamonti, Margherita Maffioli, Domenica Caramazza, Mario Cazzola

**Affiliations:** ^1^ Division of Hematology, Department of Internal Medicine, Ospedale di Circolo e Fondazione Macchi, Varese, Italy; ^2^ Division of Hematology, Department of Oncology and Hematology, Fondazione IRCCS Policlinico San Matteo, University of Pavia, Italy

**Keywords:** myelofibrosis, polycythemia, thrombocythemia, JAK2

## Abstract

Most *BCR-ABL1*-negative myeloproliferative neoplasms (MPN) carry an activating *JAK2* mutation. Approximately 96% of patients with polycythemia vera (PV) harbors the *V617F* mutation in *JAK2* exon 14, whereas the minority of *JAK2* (V617F)-negative subjects shows several mutations in exon 12. Other mutation events as *MPL*, *TET2*, *LNK*, *EZH2* have been described in chronic phase, while *NF1*, *IDH1*, *IDH2*, *ASX1*, *CBL* and *Ikaros* in blast phase of MPN. The specific pathogenic implication of these mutations is under investigation, but they may have a role in refinement of diagnostic criteria and in development of new prognostic models. Several trials with targeted therapy (*JAK* inhibitors) are ongoing mostly involving patients with PMF, post-PV MF and post-essential thrombocythemia (ET) MF. Treatment with ruxolitinib and TG101348 has shown clinically significant benefits, particularly in improvement of splenomegaly and constitutional symptoms in MF patients. On the other hand, *JAK* inhibitors have not thus far shown disease-modifying activity therefore any other deduction on these new drugs seems premature.

Chronic myeloproliferative neoplasms (MPN) include three main diseases that are polycythemia vera (PV), essential thrombocythemia (ET) and primary myelofibrosis (PMF) [1].

As illustrated in Figure 1, ET patients may slowly progress to PV, especially those carrying the JAK2 (V617F) mutation [2, 3]. Furthermore, PV and ET have a variable risk of transformation to secondary myelofibrosis (post-PV and post-ET MF) [4, 5] and subsequently to acute myeloid leukemia (AML) [6]. Finally, AML may occur directly from ET and PV without the intermediate step of MF, in which case AML may lack *JAK2* mutation even if arising from JAK2-positive MPN [7]. Evolution to post-PV and post-ET myelofibrosis occurs at a rate of 10% to 20% after 15 to 20 years of follow-up [5]. Progression to AML is less frequent in PV and ET (2-7%) than in PMF (8-30%) [2, 8-10].

**Figure 1:**
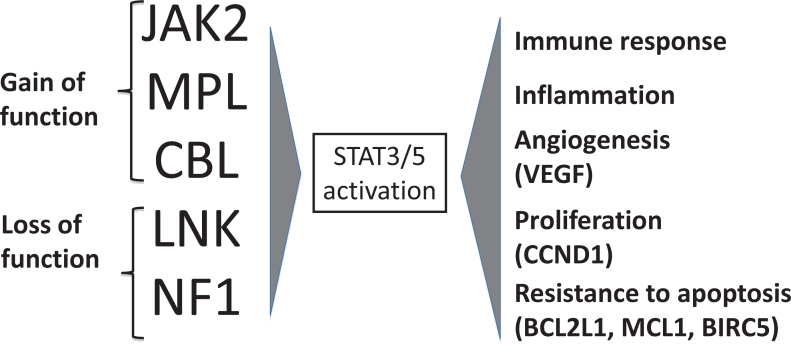
Natural history of myeloproliferative neoplasms Most frequent clinical complications in MPN patients are thrombosis, whereas hemorrhage is above all observed in essential thrombocythemia (ET) patients. ET may slowly develop into polycythemia vera (PV), especially if it carries the *JAK2* (V617F) mutation. PV and ET may progress to myelofibrosis (post-ET, post-PV MF) and then turn into acute myeloid leukemia (AML), although they may evolve into AML even without showing a MF phase.

## TOWARDS MOLECULAR UNDERSTANDING OF MPN

**Figure 2:**
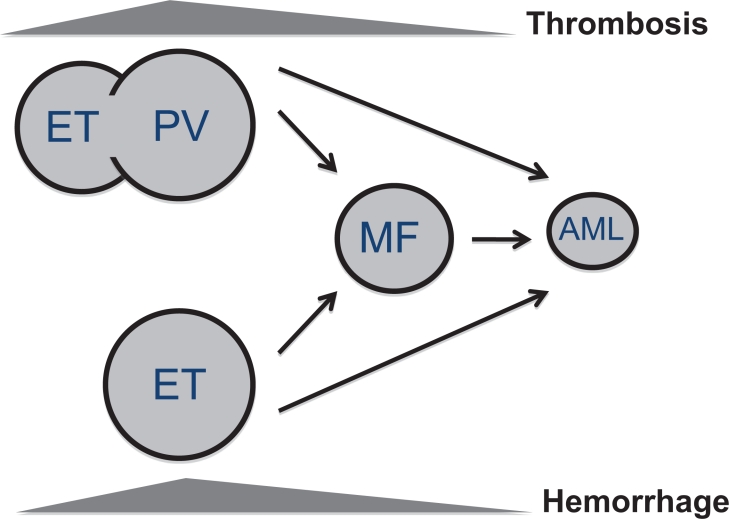
MPN mutations activating STAT3/5 Mutations of *JAK2*, *MPL* and *CBL* (due to gain of function) and mutations of *LNK* and *NF1* (due to loss of function) activate STAT3/5 which, through nuclear signal transduction, determines an amplification of immune response, inflammation, angiogenesis and proliferation, mostly modulated by higher circulating cytokines levels. STAT3/5 activation also confers resistance to apoptosis which promotes and supports myeloid precursor proliferation.

The as yet unfinished story of MPN pathogenesis started with the discovery of the *JAK2* (V617F) mutation;[11] afterwards many other mutations have been found in chronic (exon 12 mutations of *JAK2, MPL, TET2, LNK, EZH2*) and blast phase (*NF1, IDH1, IDH2, ASXL1, CBL, Ikaros*) of MPN, some involving JAK-STAT signaling activation, others chromatin remodeling and others leukemic transformation. Mutations with a gain of function of *JAK2, MPL, CBL* and those with a loss of function of *LNK* and *NF1* activate the JAK-STAT pathway[12] leading to a final phenotype of MPN with alteration of immune response, inflammation, angiogenesis, proliferation and resistance to apoptosis (Figure 2). This pathway is the target of new JAK2 inhibitors.

### Mutations mainly found during chronic phase of MPN

#### JAK2 (V617F)

*JAK2* (V617F) mutation (Janus kinase 2), occurring within exon 14 of *JAK2* and located on 9p24 is the most frequent mutation in MPN, ranging from roughly 96% in PV to 65% in ET and PMF.[11, 13] This mutation affects the auto-inhibitory domain (JH2, pseudokinase) of *JAK2* leading to constitutive activation of *JAK2* and JAK/STAT signaling. In retroviral mouse models *JAK2* (V617F) confers a PV-like phenotype with a final evolution to MF,[14] whereas when modulating allele burden, lower mutant load generates thrombocythemia and higher mutant burden results in polycythemia [15]. This means that an increased signaling through *JAK2* (V617F) may be responsible for a PV phenotype, as demonstrated in patients [13]. Clinical phenotype does not depend only on allele burden, in fact, downstream of JAK2, an enhanced phosphorylation of STAT1 or STAT5 may promote megakaryopoiesis or erythropoiesis [16].

#### JAK2 exon 12 mutations

*JAK2* exon 12 mutations have been described in *JAK2* (V617F)-negative PV and cover less than 2% of PV diagnoses [17]. Seventeen different mutations have been described with N542-E543del, K539L, and E543-D544del as the most frequent ones [18]. Exon 12 mutations result in strong ligand-independent signaling through JAK2 as demonstrated by the high levels of phospho-JAK2 and also of phospho-ERK1 and phospho-ERK2 [17], highlighting the cross talking with the Ras–ERK signaling pathway. Compared with *JAK2* (V617F)-positive PV patients, those with exon 12 mutations had significantly higher hemoglobin level and lower platelet and leukocyte counts at diagnosis but similar incidences of thrombosis, myelofibrosis, leukemia, and death [18].

#### MPL mutations

The *MPL* (myeloproliferative leukemia virus) gene, located on 1p34, can comprise different mutations within exon 10 targeting the transmembrane domain of MPL receptor [19]. The parent of these mutations is the W515L, resulting in constitutive activation of the JAK/STAT pathway. Mutation frequency is estimated at 3-5% for ET and 8-10% for PMF.[20, 21] In W515L-murine models, the mutation confers a PMF-like phenotype with thrombocytosis, splenomegaly, and fibrosis. In some instances *MPL* mutations and *JAK2* (V617F) coexist as two independent clones or two subclones [20], revealing the genetic complexity of MPN.

#### TET2 mutations

*TET2* (ten eleven translocation), a putative tumor suppressor gene located on 4q24, can be affected by an array of frameshift, nonsense and missense mutations [22, 23]. Experiments with NOD–SCID mice suggest that *TET2* might be involved in self-renewal pathways relevant to hematopoietic transformation [23]. Hierarchically, *TET2* mutations occur before or after the acquisition of *JAK2* mutations or may be an independent event [24]. In a large cohort of MPN patients, *TET2* mutations were detected in 16% of PV, 5% of ET, 17% of PMF, 14% of post-PV MF, 14% of post-ET MF and 17% of blast phase MPN; but *TET2* mutations are also described in other myeloid malignancies such as myelodisplastic syndromes (MDS), MPN/MDS syndromes and acute myeloid leukemia with variable, although not unequivocally defined, prognostic impact.

#### LNK mutations

*LNK*, located on 12q24.12, encodes for LNK, a plasma membrane-adaptor protein whose functions include inhibition of wild type and mutant JAK2 signaling [25]. In fact, LNK is a negative regulator of thrombopoietin-mediated JAK2 activation. It’s intriguing that LNK-deficient mice exhibit increased number of megakaryocytes and erythrocyte progenitors, as well as an expanded hematopoietic stem cell pool with enhanced self renewal [26]. Loss of function mutations of LNK situated within exon 2 have been described at low frequency in ET and PMF, and in erythrocytosis with low erythropoietin [27, 28].

#### EZH2 mutations

Enhancer of zeste homolog 2 (EZH2) located on 7q36.1 encodes the catalytic subunit of the polycomb repressive complex 2 (PRC2), a highly conserved histone H3 lysine 27 methyltransferase that influences stem cell renewal by epigenetic repression of genes involved in apoptosis [29]. *EZH2* has oncogenic activity. Different mutations have been found in patients with myeloid malignancies with a mutation frequency of 12% in MDS/MPN and of 13% in MF [29].

### Mutations mainly found outside chronic phase of MPN

#### NF1 mutations

*NF1* (neurofibromatosis-1) (17q11.2) is associated with the hereditary von Recklinghausen’s neurofibromatosis. It has been shown that these patients have an increased risk of developing various tumors including myeloid leukemia [30]. NF1 functions as a negative regulator of the RAS signal transduction pathway, cross-talking with the JAK-STAT pathway, and loss of NF1 can lead to a progressive myeloproliferative disorder. NF1 mutations were described in few patients with post-ET and post-PV MF, while no patients with chronic phase MPN carried these mutations [31].

#### IDH1 and IDH2 mutations

Isocitrate Dehydrogenase 1 and 2 (*IDH 1* and *IDH2*) are located at 2q33.3 and 15q26.1, respectively.[32] IDH1 mutated protein produces 2-hydroxyglutarate (2-HG). Although the role of 2-HG in tumor initiation and growth is not fully understood, this putatively oncogenic metabolite plays a role in MPN progression to leukemia besides the well defined role in the pathogenesis of gliomas [33].The frequency of these mutation in chronic MPN such as PV, ET and PMF is under 5%, but it becomes 21% in post-MPN AML [34].

#### ASXL1 mutations

*ASXL1* (Additional Sex Combs-like 1) is located on 20q11.1 and belongs to a family of three identified members that encode poorly characterized proteins regulating chromatin remodeling, enhancing transcription of certain genes while repressing the transcription of others.[35] Mutations, mainly frameshift and nonsense, occur within exon 12. Both TET2 and ASXL1 alterations lead to an increase in the program of self-renewal in MPN progenitors through modifications of DNA and histone regulation. Mutations within ASXL1 are found in 8% of MPN, 11% of MDS, 43% of chronic myelomonocytic leukemia, 7% of de novo AML, and 47% of secondary AML [35].

#### CBL mutations

The casitas B-lineage lymphoma (c-*CBL*) gene is located on 11q23.3. CBL is a well characterized protein that plays both negative and positive regulatory roles in tyrosine kinase signalling.[36, 37] CBL targets a variety of activated tyrosine kinases for degradation and may also serve as an adaptor by recruiting some downstream transduction components. Mutations in this gene are more frequent in juvenile myelomonocytic leukemia (17%) than in MPN (6% in PMF) [38].

#### IKAROS mutation

The transcription factor Ikaros encoded by the *IKZF1* gene (7p12) has a role in the regulation of hematopoiesis. In murine models, deficiency of Ikaros function may induce lymphoproliferative disorders and B- and T-cell leukemia, but also anemia and thrombocytopenia. In MPN, hemizygous loss of *IKZF1* was detected in 21% of post-MPN leukemia and in 0.2% of chronic phase MPN indicating oncogeneic properties of *IKAROS* defects [39]. Post-MPN AML involving *Ikaros* may be due to the genetic instability after *Ikaros* deletion or, alternatively, by the recently documented interaction of *Ikaros* with the JAK-STAT pathway.

## TOWARDS NEW TARGETED THERAPIES

Many drugs are now under investigations targeting different pathways critical for MPN development, such as the JAK-STAT (JAK2-inhibitors: INCB018424, TG101348, CEP701, CYT387, SB1518, AZD1480, XL019, LY2784544), the mTOR (everolimus), the MAPK (erlotinib), and the NF-Kb (bortezomib) pathways, or act through remodeling chromatin with a key role in epigenetics (givinostat, panobinostat, vorinostat). For a best update on new trials, we advise to check www.clinicaltrials.gov.

Most of JAK2 inhibitors are small molecules that act by competing with ATP for the ATP-binding catalytic site in the kinase domain. Preclinical studies have demonstrated activity of these drugs by direct inhibition of interleukin-6 signaling and of proliferation of *JAK2*(V617F)-positive Ba/F3 cells [40, 41]. In mouse models of *JAK2* (V617F)-MPN, JAK2 inhibitors markedly reduced splenomegaly and preferentially eliminated neoplastic cells, resulting in significantly prolonged survival of mice. While treatment with a JAK2 kinase inhibitor ameliorates the MPN phenotype, it does not eliminate the disease-initiating clone [42].

Taking together all available clinical data on MPN, one may conclude that JAK2 inhibitors give a benefit to patients with MF, by reducing spleen size of ~ 50% in approximately 40-50% of patients and by abolishing symptoms in the vast majority of MF patients. However, effect on these disease manifestations should be balanced with the safety profile. Anemia and thrombocytopenia are on-target toxicities expected with all JAK2 inhibitors. Other toxicities may involve non-JAK2 targets, as in case of gastrointestinal events during therapy with JAK2 inhibitors with off-target activity against FLT3 (CEP 701, TG101348, SB1518). For the current paper, we decided to report only data from the most promising JAK2 inhibitors, such as INCB018424 and TG101348, whose results are already available as full paper.

### 

#### INCB18424, Ruxolitinib

A phase I/II trial with ruxolitinib (JAK1, JAK2 inhibitor, orally bioavailable) was conducted in 152 patients with PMF or post-PV/post-ET MF [40]. Eligible subjects were therapy-requiring patients, refractory, relapsed, intolerant to previous therapy, or patients with intermediate or high-risk Lille score, if at diagnosis. Main exclusion criteria were thrombocytopenia (platelets < 100 x10^9^/L) and neutropenia (ANC <1500 x10^9^/L). The results available to date can be summarized in the following points. First, 15 mg BID (10 mg BID if platelet count <100 x10^9^/L) was the best starting dose. Second, applying IWG-MRT criteria, 44% of patients obtained a clinical improvement (CI) of spleen size (≥50% reduction from baseline) by palpation at 3 months and responses were maintained at 12 months in more than 70% of patients. The majority of patients had ≥50% improvement in constitutional symptoms due to the activity against pro-inflammatory cytokines. Among red blood cell (RBC) transfusion-dependent patients, 14% become RBC transfusion-independent. Third, no differences were reported in term of response rates according to disease type (primary or secondary MF) or *JAK2* (V617F) mutational status. Fourth, non-hematologic toxicity occurred in less than 6% of patients and was usually grade 2. At a dose of 15 mg BID, grade 3 thrombocytopenia occurred in 3% of patients and new onset of anemia in 8% of RBC transfusion-independent patients. Thrombocytopenia was more frequent if platelet count < 200 x10^9^/L at treatment start; however, this toxicity proved to be reversible.

Two randomized trials with ruxolitinib are ongoing in MF patients: COMFORT I, randomizing ruxolitinib versus placebo, and COMFORT II, randomizing ruxolitinib versus best available therapy. The primary endpoint was the number of subjects achieving ≥ 35% reduction in spleen volume from baseline to week 24 for COMFORT I and the number of subjects achieving ≥ 35% reduction in spleen volume from baseline to week 48 for COMFORT II. Media release has recently revealed that both trials have met the primary endpoint.

#### TG101348, SAR302503

A phase I trial with TG101348 (JAK2 inhibitor, orally bioavailable) was conducted in 59 patients with PMF or post-PV, post-ET MF [41]. Eligible subjects were intermediate and high-risk patients unresponsive to current treatments. Main exclusion criteria were thrombocytopenia (PLT < 50 x10^9^/L) and neutropenia (ANC <1000 x10^9^/L). The results available to date can be summarized in the following points. First, maximum tolerated dose (MTD) was 680 mg/day and dose-limiting toxicity (DLT) was a reversible and asymptomatic increase in the serum amylase level. Dose chosen for a phase II/III trial was 400 mg or 500 mg daily. Second, applying IWG-MRT criteria of response, 59% of patients achieved CI of spleen size by palpation at 6 months. The majority of patients with constitutional symptoms, fatigue, pruritus had a durable resolution without a measurable effect on cytokines. Across doses, leukocytosis and thrombocytosis were normalized at 12 months in 57% and 90% of patients. Third, no differences were reported in term of response rate according to *JAK2* (V617F) mutational status. Fourth, 39% of patients with more than 20% *JAK2* (V617F) allele burden at enrollment had a reduction of mutation load exceeding 50% at 12 months. Fifth, grade 3 to 4 hematologic adverse events included anemia (occurring in 35% of 37 patients who were not RBC transfusion dependent at baseline), thrombocytopenia (24%) and neutropenia (10%). At doses ranging between 240 mg and 520 mg, 2 of 5 (40%) RBC transfusion-independent patients became RBC transfusion-dependent and 2 of 9 (22%) had grade 3/4 thrombocytopenia. The main non-hematologic adverse events included all grades nausea (69%), diarrhea (64%) vomiting (58%), all self-limited and controlled by symptomatic treatments. Asymptomatic increase of lipase, AST, ALT, creatinine have been reported in roughly one quarter of patients.

#### CONCLUSION

The discovery of new oncogenetic mutations in MPN has enriched our knowledge in these diseases resulting in the refinement of diagnostic criteria and in potential advantages in prognostication. JAK2 inhibitors can be beneficial to patients with improvement of spleen size and constitutional symptoms. For the time being, these are the most relevant conclusions on these new small molecules with anti-JAK2 properties and any other deduction seems premature.
